# Molecular Characterization of Invasive *Streptococcus dysgalactiae* subsp. *equisimilis*, Japan 

**DOI:** 10.3201/eid2202.141732

**Published:** 2016-02

**Authors:** Takeaki Wajima, Miyuki Morozumi, Shigeo Hanada, Katsuhiko Sunaoshi, Naoko Chiba, Satoshi Iwata, Kimiko Ubukata

**Affiliations:** Author affiliations: Tokyo University of Pharmacy and Life Sciences, Tokyo, Japan (T. Wajima); Keio University School of Medicine, Tokyo (M. Morozumi, N. Chiba, S. Iwata, K. Ubukata); Toranomon Hospital, Tokyo (S. Hanada); Saitama Institute of Public Health, Saitama, Japan (K. Sunaoshi)

**Keywords:** Streptococcus dysgalactiae subsp. equisimilis, emm type, multilocus sequence typing, bacteria, Japan, streptococci

## Abstract

We collected β-hemolytic streptococci (1,611 isolates) from patients with invasive streptococcal infections in Japan during April 2010–March 2013. *Streptococcus dysgalactiae* subsp. *equisimilis* (SDSE) was most common (n = 693); 99% of patients with SDSE infections were elderly (mean age 75 years, SD ±15 years). We aimed to clarify molecular and epidemiologic characteristics of SDSE isolates and features of patient infections. Bacteremia with no identified focus of origin and cellulitis were the most prevalent manifestations; otherwise, clinical manifestations resembled those of *S. pyogenes* infections. Clinical manifestations also differed by patient’s age. SDSE isolates were classified into 34 *emm* types; *stG6792* was most prevalent (27.1%), followed by *stG485* and *stG245*. Mortality rates did not differ according to *emm* types. Multilocus sequence typing identified 46 sequence types and 12 novel types. Types possessing macrolide- and quinolone-resistance genes were 18.4% and 2.6%, respectively; none showed β-lactam resistance. Among aging populations, invasive SDSE infections are an increasing risk.

## Abstract 2

This infection is an increasing threat to aging populations.

*Streptococcus dysgalactiae* subspecies *equisimilis* (SDSE) belongs to the pyogenic group of streptococci first designated by Vandamme et al. in 1996 as a new subspecies within the species *S. dysgalactiae* (*1*). Previously isolated from humans, as commensal microorganisms, these streptococci have been designated β-hemolytic groups C and G because they are agglutinated by serum against Lancefield group C or G antigens. On blood agar plates, SDSE typically appears as large glossy colonies surrounded by a broad zone of strong β-hemolysis (*2*). For SDSE to be distinguished according to current taxonomy (*3*), specific biochemical properties need to be ascertained.

Although SDSE has long been considered much less virulent than *S. pyogenes*, many clinical and epidemiologic studies have determined that SDSE can cause a variety of severe invasive infections resembling those caused by *S. pyogenes* (*4*–*12*). These include not only cellulitis and deep abscesses but also streptococcal toxic shock syndrome (STSS) (*13*), necrotizing fasciitis, meningitis, endocarditis, and others. In addition, severity of invasive SDSE (iSDSE) infection approximates that seen with invasive *S. pyogenes* infection (*6*,*9*).

SDSE and *S. pyogenes* are considered to be closely related phylogenetically and may have originated from a common precursor (*14*). Moreover, recent genomic research has demonstrated that many pathogenically notable virulence factors in SDSE, including M protein, streptokinase, and streptolysin, were all encoded by genes highly homologous with those identified in *S. pyogenes* (*15*–*17*). However, SDSE lack several virulence factors, such as a cysteine protease (designated erythrogenic toxin B); a hyaluronic acid capsule (*hasA* and *hasB*); and an inhibitor of complement activation (*sic*) (*17*), in addition to many superantigens (*18*,*19*).

Despite this absence of some virulence factors, clinical (*4*,*13*,*20*) and epidemiologic reports (*5*,*6*,*8*–*10*,*21*) indicate that SDSE is pathogenic for humans, particularly, elderly persons with coexisting conditions. Surveillance that we conducted in 2006 implicated SDSE as a major causative pathogen in invasive β-streptococcal infections affecting the elderly in Japan (*22*). In industrialized countries, SDSE infections are frequent among elderly persons, especially among those with underlying medical conditions (*23*,*24*).

In Japan, we have organized large-scale epidemiologic surveillance for β-streptococci that are causing invasive infections and have identified SDSE as the most prevalent β-streptococcal pathogen since 2003 (*22*,*25*). However, information is limited regarding molecular characteristics of isolates and early indicators of prognosis for patients with these infections.

On the basis of *emm* genes that show polymorphisms similar to *S. pyogenes* (*26*), gene sequence analysis has been applied to *emm* typing for epidemiologic study of SDSE. According to the Centers for Disease Control and Prevention (CDC; http://www2a.cdc.gov/ncidod/biotech/strepblast.asp), >90 *emm* types have been recognized among SDSE. We previously reported that in Japan, *stG485* and *stG6792* were more prevalent in isolates from iSDSE infections, whereas *stG10* and *stG6* were more prevalent in noninvasive strains (*22*). Predominance of *emm* types also has been found to vary by geographic region.

In this study, we aimed to clarify molecular and epidemiologic characteristics of isolates from patients with iSDSE infections and the clinical features of these infections. The analysis included assessing clinical manifestations according to specific patient age group, conducting *emm* typing and multilocus sequence typing (MLST), and determining antimicrobial agent susceptibility and mechanisms of resistance to antimicrobial agents.

## Materials and Methods

### Study Design and Case Definition

We conducted nationwide surveillance of iSDSE infections during April 2010–March 2013, supported by a grant from the Japanese Ministry of Health, Labour and Welfare. After we obtained written permission from the laboratory director or hospital director, 341 general hospitals with a clinical microbiology laboratory participated in this surveillance project. Participating hospitals were located throughout Japan. Surveillance for iSDSE was carried out in parallel with 3 other investigations concerning invasive pneumococcal diseases (*27*), invasive *S. pyogenes* diseases, and invasive *S. agalactiae* diseases (*28*). 

Infections with iSDSE were defined as cases in which SDSE was isolated from normally sterile clinical samples such as blood, cerebrospinal fluid, joint fluid, or pus obtained from within a closed space. Strains were sent by the various participating institutions when SDSE was re-identified by β-hemolysis on sheep blood agar (Becton Dickinson, Tokyo, Japan) and met the following criteria: agglutination results indicated Lancefield group A, C, or G; resistance to bacitracin; lack of L-pyrrolidonyl arylamidase, according to the Manual of Clinical Microbiology (*2*); and, for some isolates, 16S rRNA sequencing results consistent with SDSE. Isolates were stored at −80°C in 10% skim milk until use (Becton Dickinson, Sparks, MD, USA).

### Requested Information

We asked attending physicians to complete and anonymously submit questionnaires along with iSDSE isolates. Requested data included patient age at onset, patient sex, origin of sample, clinical manifestation or diagnosis, underlying diseases, prior administration of antimicrobial agents, antimicrobial agent used for the infection, clinical laboratory data obtained at hospitalization, and outcome at discharge. Clinical manifestations and diagnoses were verified by a pulmonologist, according to the diagnostic criteria for sepsis based on the guidelines of the American College of Chest Physicians and the Society of Critical Care Medicine (*29*,*30*), as well as input from attending physicians, in the context of the definition of STSS established by CDC (*31*).

### *emm* Typing and MLST

Typing of the *emm* gene was performed as described (*22*,*25*), by amplification by PCR, after which resulting PCR fragments were sequenced. Each *emm* type was identified by using the CDC *emm* sequence database (http://www2a.cdc.gov/ncidod/biotech/strepblast.asp).

MLST was performed according to the method of Ahmad et al. (*32*). First, 7 housekeeping genes, *gki* (glucose kinase*)*, *gtr* (glutamine transport protein), *murI* (glutamate racemase), *mutS* (DNA mismatch repair protein), *recP* (transketolase), *xpt* (xanthine phosphoribosyl transferase), and *atoB* (acetoacetyl-coathioloase) were amplified, and all amplified DNA fragments were sequenced. Sequencing results for the 7 housekeeping genes in every strain each were assigned a sequence type (ST) by using the MLST website (http://sdse.mlst.net/). Relationships of each ST were analyzed by eBURST version 3.1 (http://eburst.mlst.net/v3/).

### Antimicrobial Agent Susceptibility

Susceptibilities to 8 oral and 7 parenteral antimicrobial agents for SDSE strains were determined by agar-dilution methods by using Mueller-Hinton agar supplemented with 5% defibrinated sheep blood. Antimicrobial agents were obtained from their respective manufacturers. We used the following breakpoints recommended by the Clinical Laboratory Standards Institute (*33*): penicillin G (susceptible, ≤0.12 μg/mL); cefotaxime (susceptible, ≤0.25 μg/mL); meropenem (susceptible, ≤0.5 μg/mL); vancomycin (susceptible, ≤1 μg/mL); clarithromycin (susceptible, ≤0.25 μg/mL; intermediate, 0.5 μg/mL; resistant, ≥1 μg/mL); clindamycin (susceptible, ≤0.25 μg/mL; intermediate, 0.5 μg/mL; resistant, ≥1 μg/mL); and levofloxacin (susceptible, ≤2 μg/mL; intermediate, 4 μg/mL; resistant, ≥8 μg/mL).

### Identification of Antimicrobial Resistance Determinants

Three macrolide-resistant genes, *erm*(A), *erm*(B), and *mef*(A), were identified in iSDSE strains by PCR methods as described (*25*,*34*). To determine fluoroquinolone resistance, quinolone-resistant determining regions of *gyrA*, *gyrB*, *parC*, and *parE,* we sequenced genes and deduced amino acid substitutions (*34*,*35*).

### Statistical Analysis

We assessed statistical significance of differences for age group and specific infectious disease, macrolide or quinolone resistance, and *emm* type. We performed χ^2^ tests or the Fisher exact test using Ekuseru-Toukei 2012 software for statistics (Social Survey Research Information, Tokyo, Japan).

## Results

### Age Distributions of Patients 

**Figure 1:**
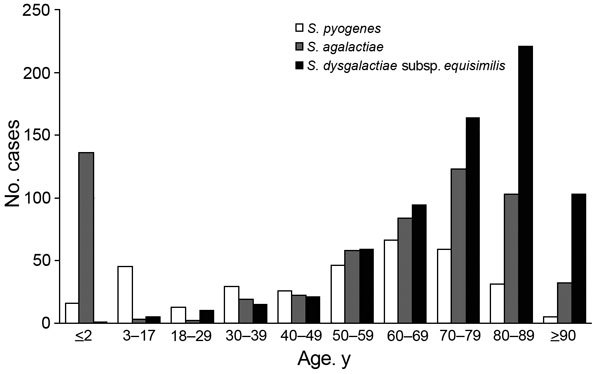
Age distribution of patients with invasive β-streptococcal infections, Japan, April 2010–March 2013. *Streptococcus pyogenes,* n = 336; *Streptococcus agalactiae,* n = 582; *Streptococcus dysgalactiae* subsp. *equisimilis,* n = 693. Means and SDs of ages in patients ≥18 years of age for each pathogen were the following: *S. pyogenes* (mean 61 years, SD ± 17), *S*. *agalactiae* (mean 70 years, SD ± 15), and *S. dysgalactiae* subsp. *equisimilis* (mean, 75 years, SD ± 15).

Age distributions of patients with invasive β-streptococcal infection caused by iSDSE, *S. pyogenes*, and *S. agalactiae* are shown in Figure 1. Infections caused by iSDSE were most prevalent (n = 693). During 3 successive periods, iSDSE infections accounted for the following numbers of cases: 231 during April 2010–March 2011, 216 during April 2011–March 2012, and 246 during April 2012–March 2013. Of all patients infected with iSDSE, 687 were adults >18 years of age (99.1%); only 6 were children. The mean age of adult patients with iSDSE infection was 75 years (SD ± 15 years), significantly older than those infected with *S. pyogenes* (61 years, SD ± 17 years) and *S. agalactiae* (70 years, SD ± 15 years) (p<0.001 for each).

### Relationships between Age Group and Clinical Manifestations

Relationships between age group and clinical manifestations in patients with iSDSE are shown in Table 1. Comorbid conditions, including a variety of underlying diseases, were found in 76.8% of patients: diabetes (22.7%), malignancies (16.7%), cardiac diseases (21.4%), and liver or renal dysfunction (16.3%). The male-to-female ratio was 1.2:1.

**Table 1:** Clinical manifestations and age of patients with invasive *Streptococcus dysgalactiae* subsp. *equisimilis* infection, Japan, April 2010–March 2013*

Clinical manifestation	No. (%) cases by age, y						Total no. (%) cases	p value†
	<18	18–59	≥60	≥70	≥80	≥90		
Cellulitis	1 (0.4)	36 (15.4)	26 (11.1)	59 (25.2)	78 (33.3)	34 (14.5)	234 (33.8)	0.702
Pneumonia		2 (4.9)	4 (9.8)	5 (12.2)	18 (43.9)	12 (29.3)	41 (5.9)	0.006
Arthritis	1 (2.1)	11 (23.4)	9 (19.1)	13 (27.7)	8 (17.0)	5 (10.6)	47 (6.8)	0.076
Abscess, noncutaneous	1 (3.2)	13 (41.9)	5 (16.1)	5 (16.1)	5 (16.1)	2 (6.5)	31 (4.5)	<0.001
Endocarditis		3 (27.3)		5 (45.5)	2 (18.2)	1 (9.1)	11 (1.6)	–
Meningitis	1	1		3		1	6 (0.9)	–
STSS		1	1	1			3 (0.4)	–
Necrotizing fasciitis	1 (6.3)	3 (18.8)	3 (18.8)	3 (18.8)	4 (25.0)	2 (12.5)	16 (2.3)	0.803
Cholangitis/peritonitis		2 (14.3)	1 (7.1)	5 (35.7)	5 (35.7)	1 (7.1)	14 (2.0)	0.740
Osteomyelitis/spondylitis		2 (14.3)	5 (35.7)	4 (28.6)	3 (21.4)		14 (2.0)	–
Bacteremia without primary focus	1 (0.4)	30 (11.1)	39 (14.4)	58 (21.5)	97 (35.9)	45 (16.7)	270 (39.0)	0.058
Others‡		1	1	3	1		6 (0.9)	–
Total	6 (0.9)	105 (15.2)	94 (13.6)	164 (23.7)	221 (31.9)	103 (14.9)	693 (100)	

*STSS, streptococcal toxic shock syndrome; –, not determined because of small number of strains. Blank cells indicate 0. †p values were calculated for differences between the 5 age groups, except the <18 y group. ‡Lymphangitis (n = 5) and keratitis (n = 1).

SDSE caused a variety of invasive infections. Most common was bacteremia without an identified primary focus (39.0%), followed by cellulitis (33.8%) and septic arthritis (6.8%); pneumonia with a positive blood culture accounted for 5.9%. STSS (0.4%) and necrotizing fasciitis (2.3%) occurred infrequently, as did endocarditis (1.6%), cholangitis/peritonitis (2.0%), and osteomyelitis/spondylitis (2.0%). Pneumonia occurred in patients >80 years of age (p = 0.006); in contrast, septic arthritis and noncutaneous abscesses tended to occur in patients <59 years of age (p = 0.076 and p<0.001, respectively).

### *emm* Type, Clonal Complex, and ST

Correlations between *emm* type and clonal complex (CC) in iSDSE strains are shown in Table 2. The *emm* types were classified into 34 groups. The most prevalent was *stG6792*, which accounted for 27.1% of isolates, followed by *stG485* (13.3%), *stG245* (10.7%), *stG652* (6.8%), *stG10* (6.2%), and *stG6* (5.5%). These 6 *emm* types accounted for 69.6% of types in all strains. Among strains typed as *stG485* or *stG245*, 6 had Lancefield group A antigen rather than C or G.

**Table 2:** Correlation with *emm* type and clonal complex among *Streptococcus dysgalactiae* subsp. *equisimilis* isolates from invasive infections, Japan, April 2010–March 2013*

*emm* type	Clonal complex, no. (%)										Total no. (%)
	CC17	CC25	CC29	CC128	CC15	CC129	ST138/153	ST78/130	Singleton	Novel ST†	
*stG6792*	183		1		1					3	188 (27.1)
*stG485*	3		50	37						2	92 (13.3)
*stG245*	1	68		5							74 (10.7)
*stG652*	6	19	7		1		3		2	9	47 (6.8)
*stG10*	2				41						43 (6.2)
*stG6*	2	26							10		38 (5.5)
*stG653*	25			1						1	27 (3.9)
*stG2078*	26										26 (3.8)
*stC36*	9					1			14	1	25 (3.6)
*stC74a*			18								18 (2.6)
*stG166b*	1	12								4	17 (2.5)
*stG480*	7				6				3		16 (2.3)
*stG5420*	1	14									15 (2.2)
*stC6979*						13					13 (1.9)
*stG4974*	10										10 (1.4)
*stG4222*	4	6									10 (1.4)
Others‡	7	4	1	2		1		2	16	1	34 (4.9)
Total	287 (41.4)	149 (21.5)	77 (11.1)	45 (6.5)	49 (7.1)	15 (2.2)	3 (0.4)	2 (0.3)	45 (6.5)	21 (3.0)	693 (100)

*ST, sequence type. Blank cells indicate 0.  †Allele numbers for novel STs are being requested. ‡Others include *stG11, stG211, stG2691, stG495, stG5345, stG97, stG643, stG62647, stC1400, stC46, stC5345, stC9431, stC10, stC839, emmG2, emmG3, stGM220,* and *stL1929*.

MLST performed for all iSDSE strains yielded 46 STs and 12 novel STs. Allele numbers are being requested for the novel STs. Novel STs accounted for 3.0% of strains.

**Figure 2:**
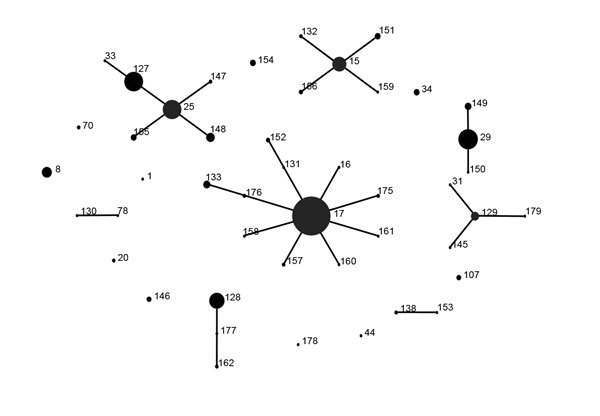
eBURST analysis (http://eburst.mlst.net/v3/) of *Streptococcus dysgalactiae* subsp. *equisimilis* from invasive infections, Japan, April 2010–March 2013. Eight clonal complexes (CC) were identified: CC15, CC17, CC25, CC29, CC128, CC129, ST78/ST130, and ST138/ST153.

Results of eBURST analysis are shown in Figure 2. These STs were classified into 8 CCs and 10 singletons. Among them, CC17 was most prevalent (41.4%, n = 287), followed by CC25 (21.5%, n = 149) and CC29 (11.1%, n = 77).

To clarify relationships between *emm* type and CC, we identified a dominant CC for each *emm* type. Except for *stG652* strains, CCs in almost all strains in *stG6792* and *stG653,* and all of those in *stG2078* and *stG4974*, were identified as CC17. Similarly, strains in *stG245, stG6, stG166b*, and *stG5420* were assigned to CC25, *stC74a* to CC29, *stG10* to CC15, and *stC6979* to CC129. Several *emm* types, *stG485*, *stG652*, *stC36*, *stG480*, and *stG4222,* belonged to >2 different CCs. No significant correlation was found between *emm* type and fatality rate for infected patients (Technical Appendix Figure 1).

### Identification of Novel *emm* Types

We identified 2 novel *emm* types, *emmG2.0* and *emmG3.0,* among iSDSE strains (Technical Appendix Figure 2). *G2.0* was a new *emm* type in which 21-bp deletions occurred in *stG245.0*, and *emmG3.0* had a chimeric structure derived from *stG3251.0* and *stG485.0.* In these novel *emm* type strains, the STs were ST33 and ST128, which belonged to CC25 and CC128, respectively. These findings suggest that strains in *emmG2.0* and *emmG3.0* were derived from those with *stG245*-CC25 and *stG485*-CC128.

### Antimicrobial Agent Resistance and *emm* Type

Relationships between macrolide resistance or quinolone resistance and *emm* type are shown in Table 3. A total of 18.5% (n = 128) of the strains showed macrolide resistance mediated by 3 genes. Resistance conferred by the *erm*(A) gene, representing inducible resistance to macrolides, lincosamide, and streptogramin B, was found in 7.9% of isolates; that arising from the *erm*(B) gene conferring constitutive macrolide resistance was found in 8.9%; and that mediated by the *mef*(A) gene conferring intermediate resistance to macrolides was found in 1.6%. Resistant strains were distributed among >10 *emm* types. In particular, 51.4% of the *stG245* strains and 44.2% of *stG10* strains showed macrolide resistance mediated by the *erm*(A) or *erm*(B) gene. These types were related significantly to macrolide resistance (p<0.001).

**Table 3:** Correlation of *emm* type and macrolide or quinolone resistance genes among *Streptococcus dysgalactiae* subsp. *equisimilis* isolates*,* Japan, April 2010–March 2013*

*emm* type	Total no. strains	No. (%) macrolide resistance			Total no. (%) resistance	p value	No. (%) quinolone resistance†		Total no. (%) resistance	p value
		*erm*(A)	*erm*(B)	*mef*(A)			*gyrA* + *parC*	*parC*		
*stG6792*	188	10 (5.3)	7 (3.7)	7 (3.7)	24 (12.8)	0.018	2 (1.1)	3 (1.6)	5 (2.7)	0.950
*stG485*	92	12 (13.0)	1 (1.1)	2 (2.2)	15 (16.3)	0.565	4 (4.3)		4 (4.3)	0.257
*stG245*	74	1 (1.4)	37 (50.0)		38 (51.4)	<0.001		1 (1.4)	1 (1.4)	0.476
*stG652*	47	6 (12.8)	1 (2.1)		7 (14.9)	0.513	3 (6.4)		3 (6.4)	0.091
*seG10*	43	10 (23.3)	8 (18.6)	1(2.3)	19 (44.2)	<0.001	1 (2.3)		1 (2.3)	0.908
*stG6*	38	2 (5.3)	1 (2.6)	1 (2.6)	4 (10.5)	0.194			0	–
*stG653*	27				0	–		1 (3.7)	1 (3.7)	0.712
*stG2078*	26	6 (23.1)	1 (3.8)		7 (26.9)	0.656		1 (3.8)	1 (3.8)	0.725
*stC36*	25	1 (4.0)	1 (4.0)		2 (8.0)	0.169			0	–
*stC74a*	18		2 (11.1)		2 (11.1)	0.415			0	–
*stG166b*	17				0	–			0	–
*stG480*	16	2 (12.5)			2 (12.5)	0.534			0	–
*stG5420*	15				0	–		1 (6.7)	1 (6.7)	0.316
*stC6979*	13	1 (7.7)			1 (7.7)	0.312			0	–
*stG4222*	10				0	–			0	–
*stG4974*	10		2 (20.0)		2 (20.0)	0.900	1 (10.0)		1 (10.0)	0.138
Others	34	4 (11.8)	1 (2.9)		5 (14.7)	0.562			0	–
Total	693	55 (7.9)	62 (8.9)	11 (1.6)	128 (18.5)		11 (1.6)	7 (1.0)	18 (2.6)	

*Dashes indicate p value not determined because of the small number of strains. Blank cells indicate 0. †Mutations in *gyrA* gene: Ser81Phe (n = 8) and Ser81Tyr (n = 3). Mutation in *parC* genes: Ser79Phe (n = 16), Ser79Tyr (n = 1), Asp83Gly (n = 1).

Fluoroquinolone-nonsusceptible strains accounted for 2.6% of isolates (n = 18). These possessed amino acid substitutions in quinolone resistance-determining regions of GyrA and ParC, encoded by *gyrA* and *parC* genes, respectively. Strains (n = 11) for which levofloxacin MICs were at least 16 μg/mL had both substitutions of Ser81Phe or Ser81Tyr in GyrA and Ser79Phe in ParC, whereas remaining strains for which levofloxacin MICs were 4–8 μg/mL had Ser79Phe (n = 5), Ser79Tyr (n = 1), or Asp83Gly (n = 1) in ParC. These fluoroquinolone-resistant strains were distributed in 9 *emm* types, including *stG6792* (n = 5) and *stG485* (n = 4).

Susceptibilities to 8 oral and 7 parenteral antimicrobial agents, among 693 iSDSE strains, are shown in Technical Appendix Table 1. Except for cefazolin and cefotiam, which were preferred by physicians in Japan, the antibacterial activity of β-lactam agents was superior; MIC for 90% of strains tested ranged from 0.004 to 0.125 μg/mL. MICs of β-lactam agents or vancomycin were not excessive for any strain.

## Discussion

We analyzed molecular characteristics of SDSE strains from invasive infections, including *emm* typing, MLST, and antimicrobial resistance determinants, together with clinical features. Molecular epidemiologic surveillance showed that the most prevalent *emm* type was *stG6792*, which has been true for iSDSE infection since 2003 (*22*,*25*). Surprisingly, this type has not been prevalent in other countries (*6*–*11*). The reason for variation in dominant *emm* type between countries remains to be determined.

MLST analysis indicated that CC17, particularly consisting of ST17, was the most prevalent CC, which was identified in a variety of *emm* types. *S. pyogenes* strains belonging to a single *emm* type usually have shown the same CC with only single- and double-locus variants (*28*). In contrast, SDSE strains included a variety of CCs (STs), a fundamental difference from *S. pyogenes* strains*.* Data reported by McMillan et al. indicated that SDSE strains belonging to *stG2078* were classified as ST17 (CC17), whereas those belonging to *stG6792* were assigned to ST4 (CC4) (*36*). These findings may indicate that CC17 in SDSE conveyed high virulence and that *emm* gene findings have recently become more apparent.

Genomic analysis suggests that SDSE obtained several virulence genes from *S. pyogenes* by horizontal transfer. Our results also show the possibility of novel *emm* types arising from recombination events among *emm* genes in SDSE, indicating that SDSE still is undergoing change. Cross-species transmission between SDSE and other streptococci suggests diversification of SDSE and evolution of highly pathogenic SDSE.

SDSE strains in this study were uniformly susceptible to β-lactam agents, and MICs of these agents were excellent, except for those of some cephalosporin agents. In contrast, macrolide resistance was found in 18.5% of strains, an increase from our previous findings (*25*). We also previously reported that macrolide resistance increased among *S. pyogenes* strains, exceeding 50% in invasive infections (*28*) and 60% in noninvasive infections (*34*). Similarly, macrolide-resistant strains may increase among SDSE strains. Although quinolone resistance was uncommon, we predict that its prevalence will increase with increasing quinolone administration.

SDSE isolates were collected at the same time as strains of *S. pyogenes*, *S. agalactiae*, and *S. pneumoniae* (*27*) for 3 years throughout Japan. The mean age of patients with iSDSE infections was greater than of those with *S. pyogenes* and *S. agalactiae* infection. As expected, the fatality rate was significantly higher for elderly patients, especially those with pneumonia, severe sepsis, septic shock, or disseminated intravascular coagulation (data not shown). Our results identify iSDSE as a common cause of community-acquired infections in an aging society. Immunologic senescence associated with aging as well as underlying diseases are suspected to contribute to risk. Differences in virulence factors between *S. pyogenes* and SDSE, including superantigens and cysteine proteases, may be key causative factors. Further clarification of the contribution of virulence factors present at onset is needed because severe SDSE infections will probably become more frequent.

In Japan, community-acquired iSDSE infections first drew attention in 2003 (*12*,*13*), when persons >65 years of age accounted for nearly 20% of the total population. According to 2013 Japanese population statistics, the segment of the population >65 years of age had exceeded 25%, with Japan becoming the highest-ranking country in terms of average life expectancy (Statistics Bureau Japan, Ministry of Internal Affairs and Communications; http://www.stat.go.jp/english/index.htm). Given the relationship between age and iSDSE, we believe that our population dynamics particularly predispose the country to increases in iSDSE infection that may not yet be present in other countries.

In conclusion, SDSE may become a global concern as a causative pathogen with the potential for high mortality rates among elderly persons with community-acquired infections, especially in industrialized countries. Global surveillance of invasive SDSE infection is needed.

Technical Appendix Susceptibilities to 15 antimicrobial agents among 693 invasive *Streptococcus dysgalactiae* subsp. *equisimilis* (SDSE) strains and relationships between outcomes of patients with invasive SDSE infection and *emm* type of the isolate and type-specific regions in 2 novel *emm* types of *emmG2.0* and *emmG3.0* identified in SDSE strains, Japan, April 2010–March 2013.
