# Supplementary material for: Molecular Characterization of Invasive Streptococcus dysgalactiae subsp. equisimilis, Japan
Source: Emerg Infect Dis. 2016 Feb;22(2):247–54. doi: 10.3201/eid2202.141732 (PMC4734521; doi:10.3201/eid2202.141732)
Supplement: Technical Appendix — Susceptibilities to 15 antimicrobial agents among 693 invasive Streptococcus dysgalactiae subsp. equisimilis (SDSE) strains and relationships between outcomes of patients with invasive SDSE infection and emm type of the isolate and type-specific regions in 2 novel emm types of emmG2.0 and emmG3.0 identified in SDSE strains, Japan, April 2010–March 2013. [file 14-1732-Techapp-s1.pdf]

Article DOI: http://dx.doi.org/10.3201/eid2202.141732

Molecular Characterization of Invasive *Streptococcus dysgalactiae* subsp. *equisimilis*, Japan

Technical Appendix Table. Susceptibilities to 8 oral and 7 parenteral antimicrobial agents among 693 invasive *Streptococcus dysgalactiae* subsp. *equisimilis*, strains

Antimicrobial agent	MIC_{50} (µg/mL)	MIC_{90} (µg/mL)	MIC range (µg/mL)
Oral			
Penicillin	0.016	0.016	0.016–0.063
Ampicillin	0.031	0.031	0.016–0.063
Amoxicillin	0.031	0.031	0.016–0.063
Cefdinir	0.031	0.031	0.016-0.125
Cefditoren	0.016	0.016	0.008-0.031
Tebipenem	0.004	0.004	0.002-0.008
Clarithromycin	0.125	64	0.016-64
Levofloxacin	1	2	0.5-32
Parenteral			
Cefazolin	0.125	0.125	0.063-0.25
Cefotiam	0.125	0.125	0.063-0.25
Cefotaxime	0.016	0.031	0.016-0.031
Meropenem	0.031	0.063	0.031-0.125
Panipenem	0.008	0.016	0.008-0.016
Vancomycin	0.5	0.5	0.25-1
Clindamycin	0.125	64	0.063-64

MIC, Minimum inhibitory concentration

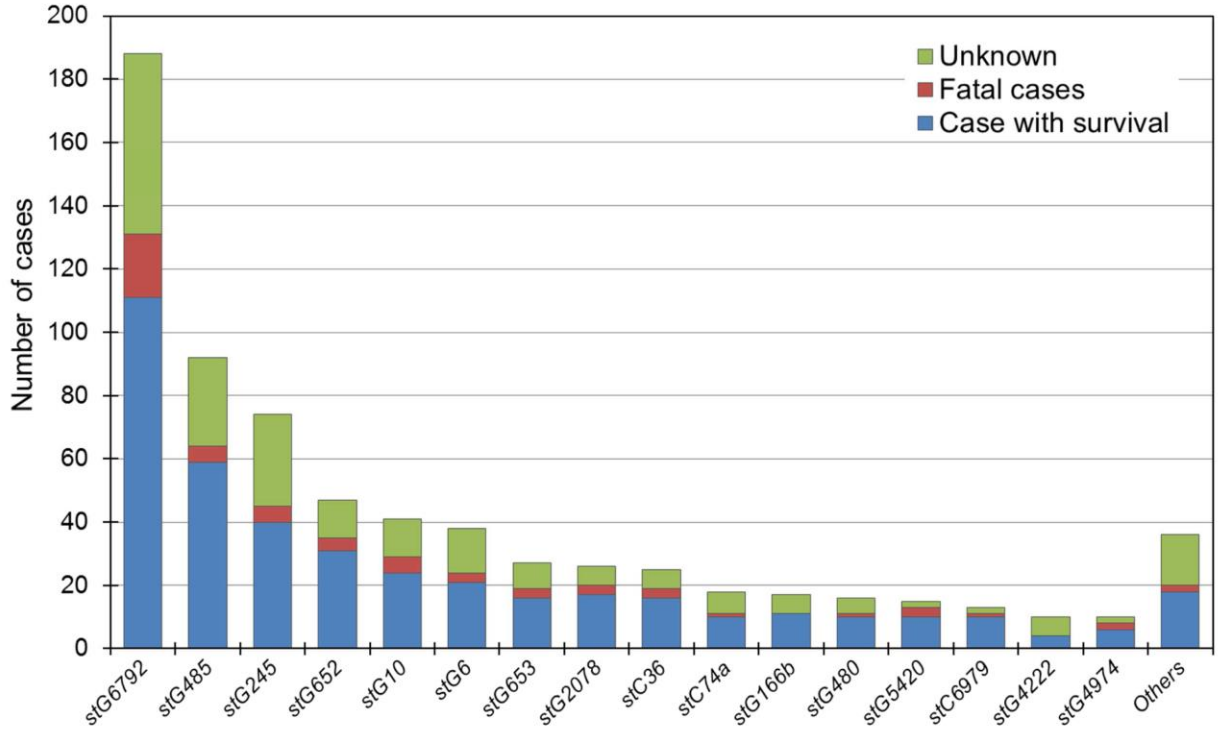

Technical Appendix Figure 1. Relationship between outcomes of patients with invasive *Streptococcus dysgalatiae* subsp. *equisimilis* infection and *emm* type of the isolate, Japan, April 2010–March 2013. No significant correlation was found (p = 0.830).

A

```
emmG2.0     GGGTTAGCAAGCCAGACAGAAGTTAAGGCGGATCATTCG--------------------- 39
stG245.0    GGGTTAGCAAGCCAGACAGAAGTTAAGGCGGATCATTCGCTAGTAGAAAACGCTAGAAAT 60

emmG2.0     AAAGTACTAAATAATAACTATATACCGTTTGAAAGGTGGCATTCGACTTATATTGAAAAA 99
stG245.0    GAAGTACTAAATAATAACTATATACCGCTTAAAAGGTGGTATTCGACTTATATTGAAAAA 120

emmG2.0     GAACGTCTTCGTAAGGAAAATGAACAACTTAACGAACTAGTTCGTAAAGAAGTTGCAGAA 159
stG245.0    GAACGTCTTCGTAAGGAAAATGAACAACTTAACGAACTAGTTCGTAAAGAAGTTGCAGAA 180
```

B

```
emmG3.0     GGGTTAGCAAGCCAGACAGAAGTTAAGGCTGAGGTTAATCACCCTAACTATACCGCTGCT 60
stG3251.0   GGGTTAGCAAGCCAGACAGAAGTTAAGGC---GGCTGAGCATCCTAGATATGTCGCTGCT 57
stG485.0    GGGTTAGCAAGCCAGACAGAAGTTAAGGCTGAGGTTAATCACCCTAACTATACCGCTGCT 60

emmG3.0     AGAAAAGACCTTAGCGATAAAA-ATCAAAAAAT---------TCAAG-AAGAGTTAGAAA 109
stG3251.0   AGA------------GATAAAA-ATCAAAAAAT---------TCAAG-AAGAGTTAGAAA 94
stG485.0    AGA------------AATGAAGTACTAAGAGATCTGTATGTATCGGGTAATATTTGGGAG 108

emmG3.0     --ACAAAAAGAAAGACTATCAGCTTGCTGTAGATGGACATGCTAATACAGTAAAACGTTA 167
stG3251.0   --ACAAAAAGAAAGACTATCAGCTTGCTGTAGATGGACATGCTAATACAGTAAAACGTTA 152
stG485.0    TGGCAAAATCAAAGA--ATTAAAGAACTACAGT------CACTTTTA-GGTAAAAC---- 155
```

Technical Appendix Figure 2. Type-specific regions in 2 novel *emm* types of *emmG2.0* and *emmG3.0* identified in *Streptococcus dysgalatiae* subsp. *equisimilis* strains, Japan, April 2010–March 2013. These alignments were generated with CLUSTAL W v. 1.83 (http://clustalw.ddbj.nig.ac.jp/).
